# Cryogels: Advancing Biomaterials for Transformative Biomedical Applications

**DOI:** 10.3390/pharmaceutics15071836

**Published:** 2023-06-27

**Authors:** Hossein Omidian, Sumana Dey Chowdhury, Niloofar Babanejad

**Affiliations:** College of Pharmacy, Nova Southeastern University, Fort Lauderdale, FL 33328, USA

**Keywords:** cryogels, biomaterials, biomedical applications, drug delivery, tissue engineering

## Abstract

Cryogels, composed of synthetic and natural materials, have emerged as versatile biomaterials with applications in tissue engineering, controlled drug delivery, regenerative medicine, and therapeutics. However, optimizing cryogel properties, such as mechanical strength and release profiles, remains challenging. To advance the field, researchers are exploring advanced manufacturing techniques, biomimetic design, and addressing long-term stability. Combination therapies and drug delivery systems using cryogels show promise. In vivo evaluation and clinical trials are crucial for safety and efficacy. Overcoming practical challenges, including scalability, structural integrity, mass transfer constraints, biocompatibility, seamless integration, and cost-effectiveness, is essential. By addressing these challenges, cryogels can transform biomedical applications with innovative biomaterials.

## 1. Introduction

Cryogels have emerged as highly versatile and promising biomaterials with numerous applications in various biomedical fields [1,2,3,4,5,6]. These highly porous interconnected structures possess unique properties such as large, interconnected pores, high surface area, elasticity, and efficient diffusion, making them ideal for pharmaceutical and biomedical applications [7,8]. Cryogels offer advantages over hydrogels, including mechanical robustness, user-friendly handling, easy storage, and sterilization capabilities [9,10,11].

The crosslinking of cryogels can be achieved through physical or chemical methods, allowing for the customization of their structure and properties. Cryogels can be classified into various types based on their properties, such as swelling behaviors, physical properties, ionic charges, and source material. In terms of swelling behaviors, cryogels can be chemically responsive, biochemically responsive, or physically responsive, depending on the stimuli they encounter. Smart cryogels exhibit dynamic and responsive behavior to environmental cues [1].

Cryogels can be prepared using copolymeric or homopolymeric interpenetrating networks, allowing for the incorporation of multiple polymers and enhancing their functionality. The ionic charges of cryogels can vary, with options such as cationic, anionic, or nonionic, influencing their interactions with molecules and environments. Cryogels can be derived from natural, synthetic, or hybrid materials, providing flexibility in terms of availability and customization. Additionally, cryogels can exhibit a range of degradability, from biodegradable to non-biodegradable, which allows for control over their lifespan and potential environmental impact [1].

In the preparation of cryogels, hydrophilic polymers are often preferred due to their favorable biocompatibility and biodegradability characteristics. Water, with a freezing temperature of 0 °C, is commonly employed as the solvent for cryogels made from hydrophilic polymers. On the other hand, for cryogels composed of hydrophobic polymers, organic solvents with suitable safety profiles are utilized, taking into consideration their respective freezing temperatures.

It’s important to note that while the use of organic solvents is feasible in cryogel preparation, there are challenges associated with residual solvent removal and reliable analytical procedures to measure the residual solvent content in the final cryogel product, especially in pharmaceutical and biomedical applications. Therefore, the choice of solvent in cryogel preparation must be carefully considered to ensure the safety and suitability of the resulting cryogel for its intended application.

The potential of cryogels in biomedical applications, including drug delivery, tissue engineering, wound healing, and antibacterial properties, has been extensively explored [12,13,14,15,16,17,18,19]. Cryogels enable the creation of macroporous hydrogels with controlled porosity, facilitating enhanced cell penetration and stability in drug delivery and tissue engineering [1,2,20].

However, the fabrication process of cryogels can be complex, and achieving desired mechanical properties for specific applications can be challenging. Despite these challenges, the unique properties of cryogels make them a promising avenue for controlled drug delivery, tissue engineering, wound healing, and antibacterial applications [12,13,14,15,16,17,18,19].

To further advance the field of cryogels, several key research directions have been identified [21]. Integration of advanced manufacturing techniques such as 3D printing or electrospinning can improve control over cryogel structure and characteristics [21]. Biomimetic cryogel design, resembling natural tissue architectures, may enhance tissue engineering and regenerative medicine [22]. Additionally, long-term stability, biodegradability, combination therapies, and drug delivery systems are areas that require further research [22,23,24]. In vivo evaluation and clinical trials are crucial to ascertain the safety and efficacy of cryogels [25].

Practical challenges, including scaling up production, maintaining structural integrity and stability, overcoming mass transfer constraints, ensuring biocompatibility, and cost-effectiveness, must be addressed for the successful implementation of cryogels in real-world applications [21,23,25].

In conclusion, cryogels offer unique properties and versatility, making them promising biomaterials for various biomedical applications [1]. Ongoing research aims to enhance cryogel fabrication, optimize their properties, and explore their potential in clinical settings [1]. Addressing the challenges and advancing the knowledge in this field will contribute to the development of innovative and effective biomaterials for biomedical applications [1,21,22,23,24,25].

This review article provides a comprehensive analysis and unique insights into the topic at hand. We have incorporated a wide range of diverse and reputable sources, including 160 references, to ensure comprehensive coverage of relevant information. Through the synthesis of findings from various studies, readers can gain a deeper understanding of the topic through a cohesive and integrated approach. Additionally, the review includes a critical evaluation of the existing literature, identifying strengths, weaknesses, and potential research gaps. Practical applications and implications of the reviewed studies are also emphasized, offering valuable guidance for researchers and practitioners in the field.

## 2. Natural Polysaccharides

### 2.1. Alginate

Alginate-based materials have found notable applications in various fields, including cancer therapy, controlled drug delivery, sustained protein release, and wound care. In the realm of cancer therapy, a research study has proposed an innovative approach utilizing injectable alginate cryogels loaded with spermine-modified acetalated dextran nanoparticles (Sp-AcDEX NPs) and Nutlin-3a [26]. This combination strategy aims to enhance the accumulation of Sp-AcDEX NPs in tumor tissue and induce immunogenic cell death through Nutlin-3a. The study suggests that this approach holds promise for cancer therapy by potentially facilitating in situ cancer vaccination and utilizing antigens and danger signals from apoptotic cancer cells.

Moreover, alginate cryogels have been investigated for controlled drug delivery applications. Researchers have introduced a novel approach incorporating gold nanorods into macroporous alginate cryogels [27]. This system enables high loading of the chemotherapeutic drug Mitoxantrone and allows for its on-demand release through near-infrared irradiation. The study demonstrates the efficacy of this approach in suppressing tumor growth in vivo, highlighting its potential application in various drug delivery systems.

Another area of advancement in alginate-based materials involves the development of injectable nanocomposite hydrogels for sustained protein release. A research study presented an injectable nanocomposite hydrogel formed through bio-orthogonal crosslinking of alginate using tetrazine-norbornene coupling [28]. The hydrogel incorporates pre-adsorbed charged Laponite nanoparticles to provide sustained protein release. The study demonstrates that the kinetics of protein release can be precisely tuned by modifying the Laponite content within the hydrogels. This approach simplifies the design of hydrogel drug delivery systems and has broad potential for therapeutic protein release applications.

**Figure 1:**
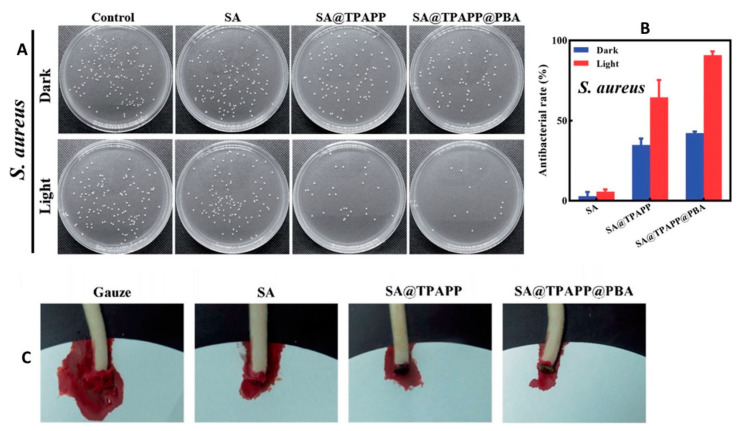
(**A**) Picture of bacterial colonies after different treatments in the presence and absence of light. (**B**) The antibacterial rates determined by the plate counting method. (**C**) Images of hemostasis by using gauze, SA, SA@TPAPP, and SA@TPAPP@PBA aerogels [29].

In the field of wound care, researchers have proposed an innovative solution utilizing an antibacterial photodynamic wound dressing composed of sodium alginate (SA), photosensitizers, and phenylboronic acid (PBA) cross-linked by Ca(II) ions [29]. This aerogel-based dressing exhibits improved solubility, hemostasis capacity, and antibacterial activity against Staphylococcus aureus. Through investigations on antibacterial activity and hemostasis, researchers found that the SA@TPAPP@PBA aerogel outperformed the SA@TPAPP aerogel (Figure 1). The reversible covalent bonds formed between phenylboronic acid and diol groups on bacterial cell surfaces enhance the aerogel’s antibacterial photodynamic therapy.

An innovative approach to neural tissue engineering is proposed in a research study utilizing an injectable neural scaffold composed of macroporous cryogels made from alginate and carboxymethyl-cellulose [30]. This scaffold offers several advantages, including surgical sterility and the use of native laminin for neural adhesion and neurite development. The primary objective is to provide protection and support to an extended living neuronal network during compression while enabling minimally invasive delivery. The scaffold exhibits mechanical meta-material behavior, combining a high local Young’s modulus for neuronal network protection with a soft macroscopic scale for easy injection.

In another study, the impact of different cross-linkers on the architecture of 3D porous scaffolds made from carrageenan and alginate is investigated [31]. The findings suggest that the ethyl (dimethylamino propyl) carbodiimide/N-hydroxysuccinimide (EDC/NHS) cross-linker is the most suitable option, resulting in a porous and interconnected structure with excellent physical and mechanical stability. This specific scaffold demonstrates enhanced cell attachment, improved cellular response, and higher metabolic activity compared to scaffolds incorporating other cross-linkers.

Furthermore, another investigation examines the effects of autoclave sterilization on naturally derived polymeric cryogels composed of alginate, hyaluronic acid, and gelatin [32]. The study reveals that these cryogels, formulated at optimized polymer concentrations, maintain their structural and physical properties after undergoing sterilization by autoclave. This discovery is significant as it offers a viable sterilization option for cryogels without compromising their properties, thereby facilitating their translation into clinical practice for biomedical applications. Autoclave sterilization proves to be a convenient and effective method for ensuring the sterility of polymeric cryogels, making them suitable for use in tissue engineering and various biomedical fields.

### 2.2. Chitosan

Hydrophobically modified chitosan cryogel: A hydrophobically modified chitosan cryogel was fabricated through reductive animation, with variations in alkyl chain length and substitution degree [33]. This cryogel exhibited optimized strength and demonstrated potential as a biomedical material. Its adsorption and release properties for hydrophobic dyes make it a promising candidate for controlled drug delivery applications.

Chitosan aerogel microparticles: Chitosan aerogel microparticles were prepared using supercritical fluid (SCF) technology and compared with those produced by freeze drying [34]. The SCF technology enabled the production of microparticles with uniform particle size and porosity, suitable for pulmonary drug delivery systems. The effects of chitosan molecular weight, polymer concentration, and tripolyphosphate concentration on drug release were also investigated.

Chitosan sponges with various features: Chitosan sponges cross-linked with glutaraldehyde were developed for healthcare applications [35]. These sponges possessed antibacterial, antioxidant, and controlled delivery properties for plant-derived polyphenols. By varying the chitosan concentration, cross-linking ratio, and gelation conditions, the sponges exhibited remarkable shape recovery, radical scavenging activity, and strong antibacterial properties against both Gram-positive and Gram-negative strains. The release of curcumin was dependent on the composition and preparation method of the sponges.

Cryogel-microparticle composite scaffold: A multifunctional biomaterial scaffold, termed cryogel-microparticle (C-MP) composite, was proposed for delivering bioactive molecules [36]. This composite incorporated microparticles (MPs) into the polymeric network of cryogels. The study demonstrated the uniform distribution of MPs within the cryogel’s pore wall and good biocompatibility with various cell lines. The C-MP composite showed potential for delivering bioactive/drug molecules to cells growing in 3D conditions.

Double cryogel system for dual drug delivery: A double cryogel system (DC) was proposed for dual drug delivery with different release kinetics to induce bone regeneration [37]. The DC system consisted of an outer layer of gelatin/heparin cryogel loaded with VEGF for initial release and an inner layer of gelatin/chitosan cryogel loaded with BMP-4 for sustained release. In vitro experiments demonstrated successful osteogenic differentiation, while in a cranial defect model, the DC system enhanced bone regeneration.

Biomaterial membrane for peripheral nerve regeneration: The proposed research investigated a biomaterial membrane for peripheral nerve regeneration using pure chitosan and nanofibrillated cellulose [38]. The membrane was mechanically strong, thermally resistant, and flexible, with a slow rate of swelling, making it suitable for surgical procedures. In vitro tests indicated non-toxicity to Schwann cells, while in vivo tests on rabbits showed ideal nerve regeneration as nerve conduits, leading to functional recovery within 15 days when combined with an autologous implant.

Chitosan-clinoptilolite 3D biocomposites as drug carriers: Chitosan-clinoptilolite 3D biocomposites were investigated as potential drug carriers [39]. The biocomposites, prepared with varying clinoptilolite content, exhibited morphological characteristics, water uptake, and drug release properties. Higher clinoptilolite content resulted in a more ordered porous structure and lower water uptake capacity, while also influencing the drug release behavior. The study demonstrated the potential of chitosan-clinoptilolite 3D biocomposites as effective drug carriers, offering controlled and sustained release profiles [39]. These biocomposites hold promise for applications in drug delivery systems, where the specific release kinetics and properties can be tailored by adjusting the clinoptilolite content.

Composite cryogels based on pectin and chitosan were developed for biomedical applications [40]. The study highlighted the dependence of cryogel properties on the structural features and physicochemical characteristics of the pectin and chitosan used. The addition of chitosan improved mechanical strength, surface morphology, degradation time, and adhesion to biological tissues. Cryogels based on apple pectin exhibited less immunogenicity, hemocompatibility, and light cytotoxicity, making them potential candidates for biomedicine applications.

PolyNIPAM thermosensitive macroporous scaffolds loaded with chitosan/bemiparin nanoparticles were developed for tissue engineering and controlled release of heparin [41]. The cryogels exhibited a highly porous structure, and the integration of nanoparticles into the macroporous system was observed. Swelling behavior of the cryogels was highly dependent on nanoparticles concentration, and the release profile of bemiparin was modulated by the presence of chitosan. The cryogels demonstrated excellent properties for application in tissue engineering and were not cytotoxic.

A composite cryogel scaffold made of chitosan, hydroxyapatite, heparin, and polyvinyl alcohol was prepared through cryogelation with glutaraldehyde [42]. The addition of polyvinyl alcohol (PVA) resulted in a more homogenous matrix structure, slowing down the formation of polyelectrolyte complexes. The scaffold efficiently immobilized bone morphogenic protein 2 (BMP-2) through electrostatic interactions and supported the differentiation of bone marrow mesenchymal stem cells into the osteogenic lineage. This scaffold showed potential for bone regeneration and tissue engineering applications.

A chitosan-based wound dressing with improved mechanical strength, decreased hydrophobicity, increased swelling ratio, and enhanced hemostatic properties was developed [43]. The semi-IPN cryogel was fabricated using Schiff’s base cross-linking between oxidized dextran and thiolated chitosan, with the addition of locust bean gum. The cryogels demonstrated good cytocompatibility, blood compatibility, and hemostatic potential, making them a promising candidate for use as a hemostatic dressing.

A physically crosslinked gel formulation of chitosan-graft-poly(N-isopropyl acrylamide) (PNIPAAm) and polyvinyl alcohol (PVA) was developed for smart delivery of an antifungal drug for mucosal applications [44]. Cryogels and formulations were characterized using various techniques, and the CS-g-PNIPAAm/PVA 75/25 hydrogel showed potential as a smart polymeric vehicle for topical applications.

pH-sensitive cryogels based on chitosan and 2-hydroxyethylcellulose were developed for drug delivery applications. The cryogels, crosslinked using H_2_O_2_ and N,N′-methylenebisacrylamide, exhibited opalescent sponge-like structures with good bioadhesive properties and pH-dependent swelling behavior. These cryogels were suitable for drug loading and release, particularly for the highly water-soluble drug metronidazole, which belongs to BCS Class I [45]. The study investigated the influence of chitosan content on the physico-mechanical properties of the cryogels and demonstrated their potential as drug delivery systems. These pH-sensitive cryogels offer a promising approach for controlled and targeted drug delivery in various biomedical applications.

An injectable cryogel microparticle (CMP) system has been studied for growth factor delivery in tissue regeneration applications [46]. The system utilizes methacrylated chitosan and chondroitin sulfate crosslinked through a radical crosslinking reaction to create a growth factor-releasing CMP. The study investigates the efficacy of recombinant human vascular endothelial growth factor (rhVEGF)-loaded CMP (V-CMP) in promoting neovascularization in vitro and in vivo, using a hindlimb ischemia mouse model. The results demonstrate a sustained release profile of rhVEGF from the V-CMP system, leading to effective tissue necrosis prevention and potential for various tissue regeneration applications.

A sustainable strategy has been proposed for fabricating chitosan-based drug delivery systems using polyelectrolyte complexes (PECs) stabilized by spontaneous interactions between chitosan and biocompatible polyanions [47]. PEC cryogels with tunable structures, morphologies, and mechanical properties are engineered through multiple-cryostructuration steps using carboxymethyl cellulose (CMC) or poly(2-acrylamido-2-methylpropanesulfonate sodium salt). These PEC cryogels exhibit impressive elasticity and toughness, making them suitable for drug delivery applications. The study demonstrates the sustainable release of anti-inflammatory drugs such as curcumin from the PEC cryogels, highlighting their potential as effective drug delivery systems.

Chitosan/xanthan gum polyelectrolyte complex (PEC) aerogel: A chitosan/xanthan gum PEC aerogel with bone-like structures and smooth surfaces, suitable for biomedical and environmental applications, was developed [48]. The aerogels exhibited meso- and macropore ranges and demonstrated thermal stability. Compared to cryogels, the aerogels had smaller total weight loss through thermal decomposition. This study highlights the potential of chitosan-xanthan gum-based aerogels in addressing challenges in biomedicine and related sciences.

Low-cost shape memory cryogel dressing for skin wounds: A low-cost shape memory cryogel dressing for skin wounds was proposed [49]. The cryogel was prepared by mixing chitosan and citric acid, and silver nanoparticles were introduced for antibacterial properties. The cryogel exhibited good mechanical properties and promoted hemostasis, blood cell adhesion, and wound healing in a full thickness wound model infected with *S. aureus*. This method offers a simple and effective solution for skin wound healing.

Autoclaved chitosan cryogels for wound dressing: Physically crosslinked chitosan cryogels were developed for wound dressing applications [50]. Autoclave sterilization was tested and found to enhance the resistance of the cryogels to enzymatic degradation. The autoclaved chitosan cryogels retained their biological properties and showed potential as practical wound dressings for moist wound healing.

Macroporous hydrogels for controlled release of macromolecular drugs: Macroporous hydrogels for controlled release of macromolecular drugs were designed using cryogelation [51]. The hydrogels, composed of methacrylic acid and either acrylamide or 2-hydroxyethyl methacrylate, were further modified with chitosan and PEGDGE to form interpenetrating polymer networks (IPNs). The IPNs exhibited different loading and release behavior for lysozyme, indicating their potential as effective drug delivery systems.

Low-cost cryogel wound dressing with photothermal therapy: A low-cost cryogel wound dressing composed of chitosan/silk fibroin scaffold and tannic acid/ferric ion was developed as a stimuli-responsive agent for photothermal therapy [52]. The cryogel demonstrated good flexibility, recoverability, hemostatic performance, and antibacterial activity against both Gram-negative and -positive bacteria. It also promoted cell proliferation and accelerated wound healing in animal experiments, making it a promising material for clinical wound dressings.

Tissue-engineered cryogel scaffolds for cartilage repair: Tissue-engineered cryogel scaffolds for repairing articular cartilage injuries were investigated [21]. The scaffolds, fabricated using cryogelation technology, included chitosan–gelatin–chondroitin sulfate for articular cartilage and nano-hydroxyapatite–gelatin for subchondral bone. A novel bilayer cryogel mimicking the osteochondral unit was also designed. The study evaluated biocompatibility, efficacy, and therapeutic approaches using chondrocyte exosomes and cryogel extract for osteochondral repair.

Alpha-ketoglutarate delivery for tissue engineering applications: A method for delivering alpha-ketoglutarate (alpha-KG) to cells in tissue engineering applications was proposed [53]. The study compared the efficacy of microspheres, microsphere-incorporating scaffolds, and exogenous supply for delivering alpha-KG. The results indicated that exogenous supply of alpha-KG yielded better results for fast proliferating cells, while microspheres or microsphere-incorporated scaffolds showed potential for slow-growing cells. The study also characterized chitosan and gelatin microspheres and evaluated their impact on cellular proliferation and ammonia levels in a 3D setup. This research provides insights into effective alpha-KG delivery strategies for tissue engineering applications.

Researchers have conducted several studies to explore the development and application of chitosan-based materials in the biomedical field, particularly in controlled drug delivery. One study [33] focused on creating a hydrophobically modified chitosan cryogel through reductive animation, with variations in alkyl chain length and substitution degree. The aim was to optimize the cryogel’s strength and its ability to adsorb and release hydrophobic dyes. The resulting cryogel showed significant potential as a biomedical material and a promising candidate for controlled drug delivery.

In another study [34], chitosan aerogel microparticles were prepared using supercritical fluid (SCF) technology and compared to those produced by freeze drying (FD). These microparticles were found to be suitable for pulmonary drug delivery systems. The study also investigated the influence of chitosan molecular weight, polymer concentration, and tripolyphosphate concentration on drug release.

In a separate study [35], researchers developed chitosan sponges cross-linked with glutaraldehyde, exhibiting antibacterial, antioxidant, and controlled delivery properties for plant-derived polyphenols. The composition and preparation method of the sponges influenced the release of curcumin. The sponges demonstrated shape recovery after compression, radical scavenging activity, and antibacterial properties against Gram-positive and Gram-negative strains, making them suitable for healthcare applications.

**Figure 2:**
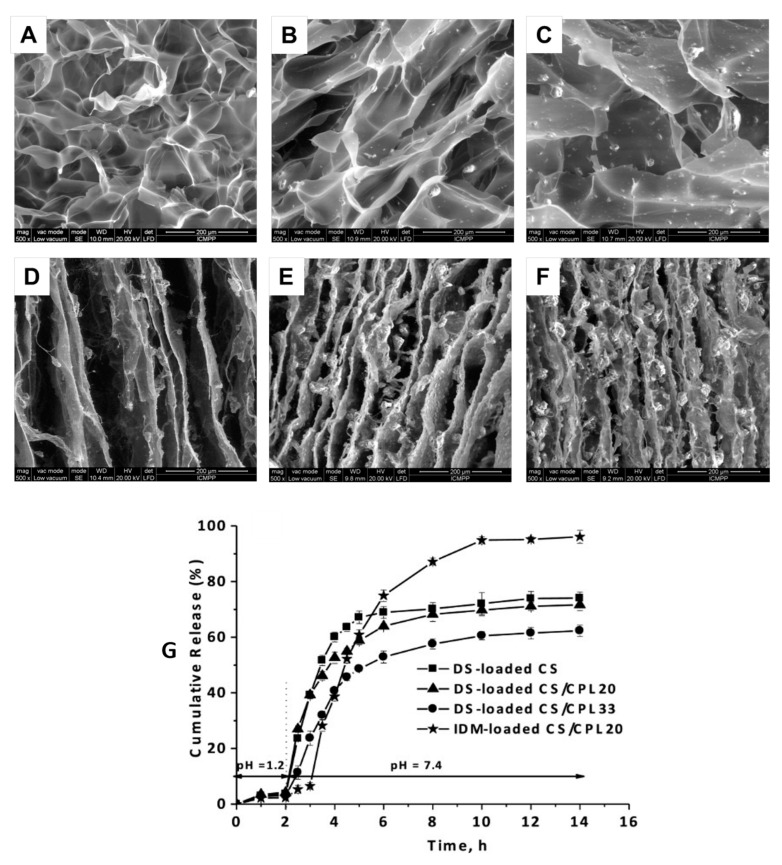
SEM images (magnification 500×) of CS/CPL composite cryogels: (**A**) only CS, (**B**) CS/CPL20, (**C**) CS/CPL33, (**D**) CS/CPL50, (**E**) CS/CPL67, and (**F**) CS/CPL80. (**G**) In vitro cumulative drug release of DS from cross-linked CS, CS/CPL20 and CS/CPL33 composite cryogels comparative with cumulative release data of IDM from CS/CPL20 composite cryogels. A total of 100 mg drug/g of cryogel was loaded. Adopted with permission from [39].

Another investigation [39] explored the potential of chitosan–clinoptilolite (CS-CPL) 3D biocomposites as drug carriers. The study observed that a higher content of clinoptilolite resulted in biocomposites with a more ordered porous structure and lower water uptake. Drug release studies using diclofenac sodium (DS)- and indomethacin (IDM)-loaded and -unloaded CS-CPL composites were conducted at two different pH levels (pH 1.2 and pH 7.4) (Figure 2). The results indicated that phosphate buffer saline was a superior medium for drug delivery compared to simulated gastric fluid, highlighting the potential of chitosan-clinoptilolite biocomposites as effective drug delivery systems.

### 2.3. Gelatin

Various studies have extensively explored the potential applications of gelatin-based cryogels in addressing osteoporosis, osteomyelitis, and tissue engineering. In one study, cryogels embedded with CaCO_3_ microspheres and ciprofloxacin hydrochloride demonstrated sustained drug release for 21 days, with enhanced cell viability and alkaline phosphatase levels, indicating their therapeutic potential for osteoporosis and osteomyelitis treatment [54]. Another study developed a cryogel nanocomposite scaffold incorporating Usnic acid encapsulated in Rhamnolipid biosurfactant nanoparticles, exhibiting improved mechanical and biological properties for osteomyelitis treatment and bone regeneration [55].

Injectable cryogels made of gelatin methacryloyl and poly(ethylene)glycol demonstrated high cell retention ability, cytocompatibility, and shape retention after strain cycles, making them suitable for cell and bioactive factor delivery [56]. Polyelectrolyte multilayer microcapsules were employed for controlled release of transforming growth factor-beta 1 (TGF-beta 1) without compromising its bioactivity, enhancing tissue repair when incorporated into gelatin-based hydrogels and cryogel scaffolds [57]. Preformed injectable cryogels composed mainly of gelatin, along with nanocarriers like cellulose nanocrystals and PAMAM dendrimers, showed potential as minimally invasive drug delivery systems in tissue engineering [58]. Furthermore, seeding human adipose-derived mesenchymal stem cells within biodegradable gelatin microcryogels resulted in tissue-like ensembles with enriched extracellular matrices and improved cell–cell interactions, demonstrating superior therapeutic efficacy for critical limb ischemia compared to free cell-based therapy [59]. Additionally, gelatin microcryogels loaded with human adipose-derived stem cells enhanced the secretion of growth factors and accelerated wound healing in vivo, providing a potential therapeutic strategy for refractory wounds [60]. Moreover, cryogenic gelatin–hyaluronic acid composite materials enabled the formation of elastic scaffolds supporting cell attachment, viability, and proliferation through 3D bioprinting [61].

Several studies have investigated the potential of cryogels as carriers for controlled drug delivery, showcasing their versatility in diverse applications. A composite cryogel combining nanocellulose and gelatin demonstrated adjustable pore structure and sustained release of the drug 5-FU, indicating its efficacy as a drug delivery system [62]. Cryogels incorporating gelatin methacrylates and nanosilicates exhibited mechanical robustness and slower degradation rates, enabling prolonged drug release under physiological conditions [63]. Preformed injectable cryogels composed of gelatin and incorporating cellulose nanocrystals and PAMAM dendrimers showed promise for minimally invasive drug delivery in tissue engineering [58]. Cryogels made of gelatin and ascorbic acid promoted enhanced metabolic activity, biosynthetic capacity, and matrix regeneration, making them suitable carriers for corneal keratocyte growth [64]. A degradable molecularly imprinted cryogel synthesized for pH-responsive delivery of doxorubicin demonstrated sustained drug release under acidic conditions [65]. Lastly, a hybrid scaffold composed of cryoelectrospun poly(epsilon-caprolactone) surrounded by a macroporous gelatin/heparin cryogel enhanced osteogenic differentiation and supported bone formation, showcasing its potential for bone tissue engineering [66].

Studies have also explored the potential of cryogels as versatile delivery systems for cells and growth factors in the fields of ischemic disease and tissue engineering. For instance, an injectable cryogel composed of gelatin and heparin facilitated effective angiogenic responses when used as a carrier for vascular endothelial growth factor (VEGF) and fibroblasts in the treatment of hindlimb ischemic disease [67]. The controlled release of VEGF from the cryogel, achieved by controlling its mechanical properties, porosity, and elasticity, promoted angiogenesis, a critical process for tissue revascularization.

**Figure 3:**
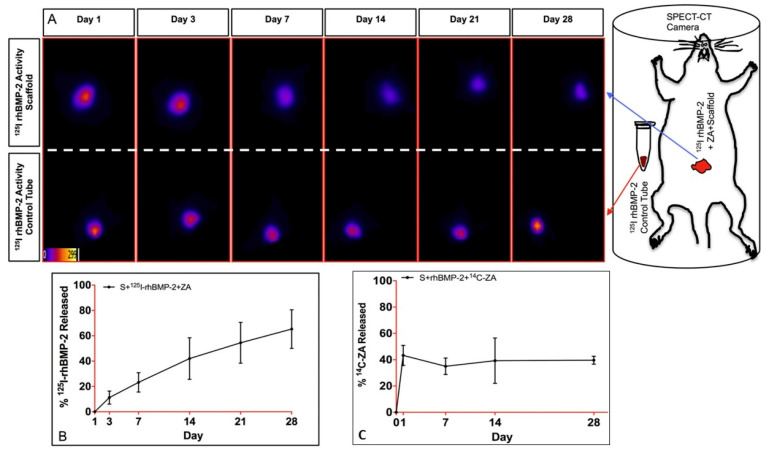
In vivo ^125^I–rhBMP-2 release from the scaffolds in the abdominal muscle pouch model during 4-weeks via SPECT (single photon emission computed tomography)imaging. (**A**) SPECT signal from the scaffold containing ^125^I–rhBMP-2 + ZA in the abdominal muscle pouch (top (**A**)) and control tube containing known amount of ^125^I–rhBMP-2 placed outside the animal almost parallel to the implant (bottom (**A**)) at different time points. (**B**,**C**) % release kinetics of ^125^I–rhBMP-2 and 14C–ZA from the scaffold in the abdominal muscle pouch during the 4-week period. Adopted with permission from [68].

Another study investigated a macroporous composite biomaterial designed for the delivery of bone morphogenetic protein-2 (rhBMP-2) and zoledronic acid (ZA) [68]. This biomaterial demonstrated superior osteogenic differentiation and mineralization compared to an approved absorbable collagen sponge. The sustained release kinetics of rhBMP-2 and ZA from the cryogel scaffold were optimized to support optimal bone healing, showing its potential in bone tissue engineering (Figure 3).

In the field of cartilage tissue regeneration, cryogels have also been explored as gene delivery and tissue engineering approaches. One study utilized cryogels to implant genetically modified chondrocytes expressing plasmid-encoding bone morphogenetic protein-7 (BMP-7) [69]. This approach resulted in enhanced matrix synthesis, chondrocyte growth, and showed potential for promoting cartilage healing.

### 2.4. Cyclodextrine

Cryogels have generated significant interest in the field of drug delivery due to their exceptional properties and diverse applications. A particular study investigated superporous p(beta-CD) cryogels with varying crosslinker ratios and identified their favorable characteristics for drug delivery [70]. The cryogels demonstrated biocompatibility, as evidenced by low hemolysis and blood coagulation indexes. Moreover, they exhibited impressive swelling and degradation properties, indicating their ability to maintain structural integrity while accommodating drug molecules. Remarkably, these cryogels showcased sustained release profiles for both hydrophilic and hydrophobic drugs, making them versatile delivery systems. Furthermore, their ability to load and release drugs simultaneously further enhances their potential for controlled drug delivery [70]. Similarly, cryogels derived from beta-cyclodextrin-modified wood-based cellulose nanofibrils (CNFs) exhibited promise for drug delivery and tissue engineering applications [71]. The modification of CNFs resulted in controlled mechanical properties and improved water sorption, facilitating efficient drug encapsulation within the cryogels. The capacity of beta-cyclodextrin to form inclusion complexes with active ingredients or growth factors further enhanced drug loading and release capabilities, expanding the potential of these cryogels in biomedical applications [71].

Another intriguing area of cryogel research revolves around enhancing the solubility and therapeutic activity of poorly soluble drugs. In a specific study, cryogel carriers incorporating beta-cyclodextrin moieties were developed for the delivery of aripiprazole, a drug with limited solubility. These carriers exhibited improved solubility and enhanced therapeutic activity of aripiprazole. By optimizing the cryogels’ DMA/beta-CD-Ac-3 mass ratio, it was possible to tailor their drug loading efficiency and release profiles, highlighting their potential as effective carriers for poorly soluble drugs [72]. Similarly, a research [73] focused on the development of super-macroporous cryogels composed of 2-hydroxyethyl cellulose (HEC) and beta-cyclodextrin (beta-CD) to enhance the solubilization and therapeutic activity of cannabidiol (CBD). The cryogels demonstrated sustained release behavior, making them potential candidates for the localized and controlled treatment of cutaneous neoplastic diseases.

Furthermore, cryogels have been investigated for antibiotic delivery. One particular research study examined a cryo-induced hydrogel composed of cellulose, carboxymethyl cellulose (CMC), and beta-cyclodextrin (beta-CD). This hydrogel exhibited high swelling properties and successful loading and release capabilities, particularly for tetracycline at pH 7.4. Additionally, the hydrogel displayed an inhibitory effect on common bacteria strains, including Staphylococcus aureus, Escherichia coli, and Pseudomonas aeruginosa. These findings indicate that the E-CMC-CEL hydrogel shows promise as a suitable material for antibiotic drug delivery platforms, providing an effective means to combat bacterial infections [74].

### 2.5. Agarose, Carrageenan, Locust Bean Gum, Natural Polysaccharides Blends

Hydrophobically Modified Agarose Cryogels: In an innovative approach, hydrophobically modified agarose cryogels were introduced as potential drug delivery systems. The modification of agarose with hydrophobic properties resulted in cryogels that displayed low cytotoxicity, reduced adhesiveness, and enhanced dye adsorption capacity compared to unmodified cryogels. Moreover, the release of the dye from these cryogels could be precisely controlled, highlighting their potential as effective drug delivery systems [75].

Leaf-Inspired Micropump: A research study presented a leaf-inspired micropump (LIM) composed of a thermo-responsive stomata-inspired membrane (SIM) and a mesophyll-inspired agarose cryogel (MAC). This smart microfluidic device demonstrated a durable flow rate and the capability to autonomously adjust the delivery rate of therapeutic liquid in response to temperature changes. The LIM shows promise as an advanced drug delivery device due to its ability to maintain a consistent flow rate and adapt to changing conditions [76].

Cactus Root-Inspired Material: A study discusses the development of a cactus root-inspired material (CRIM) incorporating cellulose fibers, microparticles, and agarose-based cryogels. The CRIM exhibits excellent water absorption and retention abilities, high structural stability, and potential applications in various fields, including cosmetics, healthcare products, functional fabrics, and drug delivery devices. The CRIM offers a straightforward and efficient solution for water management without the need for complex chemical synthesis or surface modification [77].

Agarose-Based Biomaterials in Tissue Engineering: A review article highlights the potential of agarose-based biomaterials for tissue engineering applications. Agarose, a natural polysaccharide polymer, possesses unique characteristics such as excellent biocompatibility, thermo-reversible gelation behavior, and similarities to the extracellular matrix. The review summarizes recent research on agarose-based biomaterials, emphasizing their potential in cell growth and controlled drug delivery for tissue engineering applications [78].

Alpha-Aminophosphonates in Carrageenan Cryogels: A study [79] presents a novel synthesis method for alpha-aminophosphonates with antimicrobial activity and their incorporation into carrageenan cryogels. The cryogels exhibit sustainable release behavior and demonstrate antimicrobial activity against Staphylococcus aureus. This research opens up possibilities for the use of carrageenan cryogels as carriers for antimicrobial agents, offering sustained release capabilities and potential applications in combating pathogenic microbes.

Transdermal Drug Delivery System with Kappa-Carrageenan: A study [80] proposes the use of kappa-carrageenan (kappaC) as a drug matrix material for a transdermal drug delivery system for Metformin. Porous kappaC matrices with adjustable pore sizes are fabricated, and drug release experiments reveal the potential for controlled release. Additionally, the application of an electrical potential enhances drug release–permeation. The proposed kappaC matrix holds promise for the development of transdermal controlled delivery patches for conditions such as abdominal obesity and diabetes.

**Figure 4:**
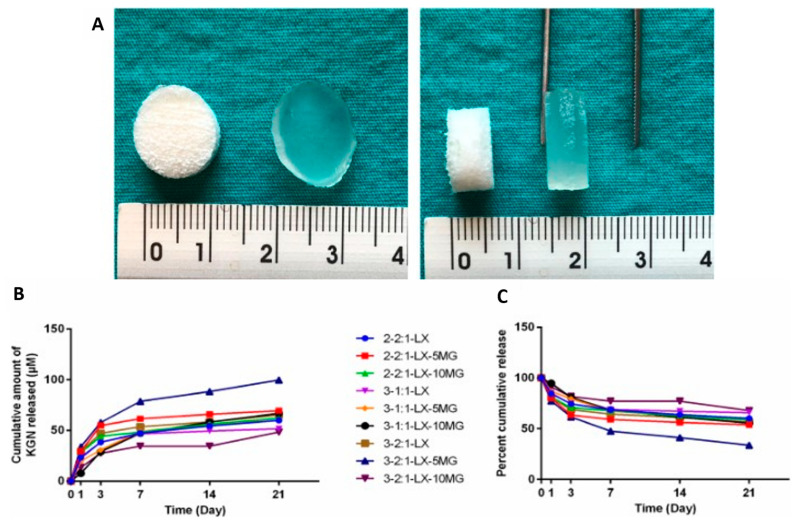
(**A**) Images of dry and wet circular cryogels, (**B**) cumulative amount of KGN release, and (**C**) percent cumulative KGN release from LX and LXM cryogels. Adopted with permission from [81].

Natural Biomaterial-Based Cryogels for Tissue Engineering and Drug Delivery: A study [81] focuses on the combination of natural biomaterials Locust bean gum, Xanthan gum, and Mastic gum (LBG, XG, and MG) to create cryogel scaffolds with macroporous and interconnected pore structures. These cryogels exhibit promising properties for cartilage and soft tissue engineering, as well as drug delivery applications. The sustained release of the small molecule Kartogenin is achieved over a period of 21 days, and the study demonstrates that the release of Kartogenin varies with different cryogel compositions (Figure 4).

Cellulose-Based Cryogels and Nanocomposite Cryogels: Several studies [22,82,83,84] explore the development and applications of cellulose-based cryogels and nanocomposite cryogels. Reference [82] introduces a novel method for creating cellulose microspheres through ethanol–hydrochloric acid pulp pretreatment, enabling diverse applications such as drug delivery systems, protein conjugation, and wastewater treatment. Reference [22] emphasizes the biocompatibility of cellulose and its utilization in biomedical materials through cryogelation. It investigates factors influencing cryogel structure and properties, and discusses applications in wound healing, tissue regeneration, and drug delivery. Reference [83] focuses on the synthesis of temperature-sensitive polymer-modified cellulose nanofibril cryogel microspheres for controlled drug release, highlighting their desirable temperature response and controllable drug release rate. Reference [84] concentrates on the development of Curcumin-nanostructured lipid carrier-loaded oleogels using biopolymer cryogels, demonstrating their improved encapsulation efficiency and control over lipolysis of lipid droplets. Collectively, these studies underscore the versatility and potential of cellulose-based cryogels and nanocomposite cryogels in various applications.

Composite Cryogels for Sustained Drug Delivery: References [85,86] address the development of cryogels for sustained drug delivery. Reference [85] proposes nanocomposite cryogels for sustained topical delivery of hydrophobic natural substances such as cannabidiol (CBD). It compares the sustained release profiles achieved by nanocomposite cryogels with those of pure 2-hydroxyethyl cellulose (HEC) cryogel carriers, demonstrating the superior performance of the nanocomposite cryogels. Reference [86] focuses on the preparation of PVP/NaCMC microspheres loaded with the antibiotic Mupirocin (MP). The study investigates various parameters affecting drug release and achieves an optimized drug release profile in a microsphere-cryogel composite system. These studies highlight the potential of cryogels as carriers for sustained drug delivery, with specific applications in topical delivery and antibiotic release.

## 3. Synthetic and Other Polymers

### 3.1. Peg Derivatives

**Figure 5:**
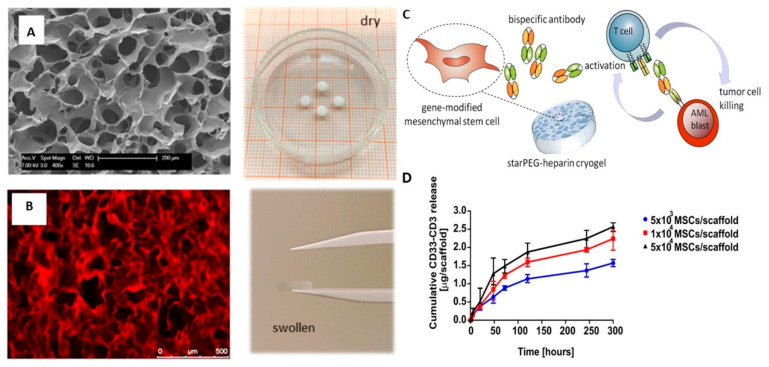
(**A**) SEM image of PEG-heparin cryogel (**left**) and photograph of the cryogel scaffold in the dry state (**right**). (**B**) Confocal laser scanning microscopy image (**left**) and photograph of cryogel scaffold after swelling in PBS (**right**). (**C**) Illustration of the cryogel-housed scBsAb-releasing MSC system. (**D**) Release profile of bsAb CD33-CD3 from modified MSCs [87].

One study focused on developing an immunotherapeutic organoid for the treatment of acute myeloid leukemia (AML) [87]. The researchers utilized a biocompatible poly(ethylene glycol)–heparin cryogel scaffold to support gene-modified mesenchymal stromal cells (MSCs) secreting an anti-CD33-anti-CD3 bispecific antibody (bsAb) (Figure 5). This approach effectively stimulated T-cell-mediated anti-tumor responses, resulting in the regression of CD33(+) AML blasts. These findings highlight the potential of cryogels in innovative immunotherapeutic strategies.

In controlled release applications, cryogels have been explored as drug reservoirs with slow-releasing properties. In one study, PEG-based bulk hydrogels and cryogels with activated carbonate groups were proposed [23]. The highly porous structure and increased swelling capacity of cryogels led to a seven-fold enhancement in drug release compared to hydrogels. Another study focused on highly porous cryogel microscale scaffolds capable of loading and releasing nerve growth factors [88]. These cryogels, with their elongated shape, successfully induced neurite outgrowth in cell cultures, demonstrating their potential for controlled release applications.

In tissue engineering, cryogels have shown promise in delivering signaling proteins and mechanically preconditioning cells. One study designed starPEG–heparin cryogel systems for the tunable, long-term delivery of signaling proteins [24]. These cryogels, with their macroporous structure, enabled precise delivery and induced neuronal differentiation of cells, making them suitable for tissue engineering applications. Another study used syringe-based 3D culture systems with poly(ethylene glycol) diacrylate microcryogels for mechanical preconditioning of mesenchymal stromal/stem cells (MSCs) towards nucleus pulposus (NP)-like cells [89]. Mechanical stimulation of the microcryogels enhanced the expression of chondrogenic marker genes, suggesting their potential in improving tissue function, particularly in intervertebral disc regeneration.

### 3.2. Polyvinyl Alcohol (PVOH)

Cryogel nanocomposites have emerged as a promising platform for drug delivery applications. In one study, a cryogel nanocomposite was developed using poly(vinyl alcohol) (PVA), zinc oxide nanoparticles (ZnO NPs), and fulvic acid (FA) [90]. This nanocomposite demonstrated well-distributed ZnO NPs and controlled drug release properties, showcasing its potential as a drug delivery system. Another study explored the thermochromic properties of PVA cryogel, which exhibited sensitivity to specific temperature ranges [91]. This versatile and cost-effective material holds promise for applications in ultrasound therapy phantoms and temperature estimation during thermal therapy.

Enhanced solubility and drug release have been investigated using PVA cryogels. In one study, the interaction between Simvastatin (SV) and PVA in cryogel matrices led to improved solubility of SV [92]. This suggests that PVA cryogel could serve as a prolonged-release matrix for hydrophobic drugs. Another study developed highly porous composite PVA cryogels loaded with poly(3-hydroxybutyrate) (PHB) microbeads containing the drug simvastatin (SVN) [93]. These composite cryogels exhibited rigidity and showed promise for controlled drug delivery.

PVA cryogels find applications in the biomedical field as well. One research proposal highlighted the versatility of PVA cryogel as a hydrogel for investigating iontophoretic transdermal drug delivery [94]. Another study focused on the development of a PVA adhesive film loaded with nanostructured lipid carriers for transdermal treatment of psychiatric disorders [95]. Additionally, PVA hydrogels and cryogels have been explored in various biomedical and medical device applications, including orthopedic and cardiovascular devices [96].

The release of DNA from PVA cryogels has also been investigated. One research proposal examined factors influencing the release rate of DNA from PVA hydrogels, such as electrolyte type, ionic strength, temperature, and surfactants [97]. Another study explored the impact of anions/cations and surfactant properties on DNA release from PVA-DNA gel matrices [98]. These findings provide valuable insights for the development of controlled DNA release systems utilizing PVA-based cryogels.

### 3.3. PVOH Hybrids

Numerous studies have been conducted on cryogels and hydrogels, focusing on their synthesis, characterization, and applications across various fields. In one study, researchers proposed a novel method for synthesizing a macroporous molecularly imprinted cryogel for solid-phase extraction (SPE) of propranolol [99]. This cryogel exhibited remarkable selectivity, stability, and regenerability, positioning it as a promising candidate for extracting target molecules from complex samples. Another study explored the preparation and evaluation of porous hydrogels made from poly(vinyl alcohol) (PVA) and gelatin using the freezing–thawing method [100]. Factors like PVA and gelatin concentration, freeze–thaw cycles, and drying temperature influenced the hydrogels’ water sorption capacity and biocompatibility. Similarly, physically cross-linked hydrogels composed of PVA and egg albumin, prepared through cyclic freezing/thawing processes, were investigated in another study [101]. These cryogels demonstrated notable biocompatibility, biodegradability, and high water sorption and swelling properties, making them suitable for various biomedical applications.

**Figure 6:**
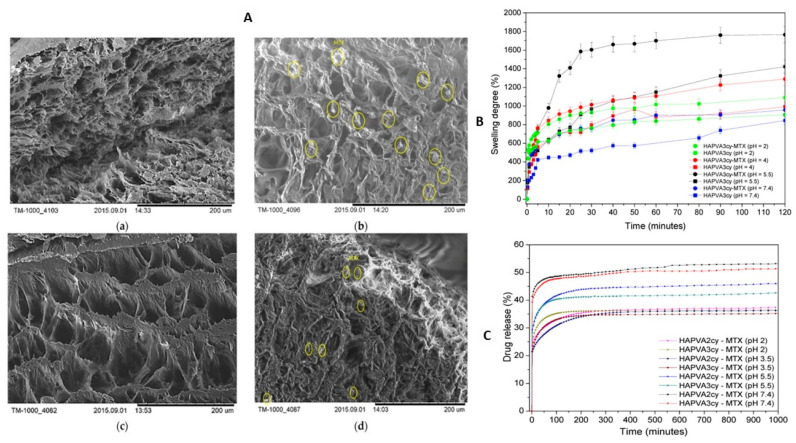
(**A**) SEM images of (**a**) HA/PVA 2c; (**b**) HA/PVA/MTX 2c; (**c**) HA/PVA 3c; (**d**) HAPVA/MTX 3c. (**B**) Swelling profiles at different pH values of unloaded and MTX-loaded HA/PVA-based cryogel. (**C**) In vitro release profile of MTX from HA/PVA-based cryogel in different pHs [103].

In the realm of drug delivery, researchers have directed their attention towards the development of smart hydrogels. One study examined a pH-sensitive hydrogel composed of a blend of polyvinyl alcohol, polyacrylic acid, and synthetic hydroxyapatite [102]. Incorporating hydroxyapatite enhanced mechanical strength, bioactivity, and drug release profiles, highlighting the potential of this smart hydrogel for targeted drug delivery. Another research proposal suggested employing poly(vinyl alcohol)/hyaluronic acid cryogels loaded with methotrexate as a pH-responsive drug delivery system for treating psoriasis [103]. These cryogels exhibited favorable physical–chemical properties, biocompatibility, reduced toxicity, and their swelling and drug release behavior responded to changes in pH (Figure 6), making them suitable for treating affected skin in psoriasis.

Furthermore, the use of composite cryogels and dual network hydrogels for controlled release applications has garnered significant interest. A novel composite cryogel was developed for the controlled release of Enalapril Maleate (EM) in the treatment of hypertension and heart failure [104]. These cryogels, composed of biodegradable polymers and zeolite L, exhibited prolonged release compared to cryogels containing only polyvinyl alcohol (PVA). In another study, tough dual network hydrogels were formulated using green chemistry and low-cost methods for potential biomedical applications [105]. These hydrogels displayed flexibility, ductility, and porosity, making them suitable for tissue matrices and customizable drug delivery systems with desired mechanical properties and physical porosity.

Lastly, a research proposal suggested utilizing a cryogel carrier system for the controlled release of extracted propolis, aiming to enhance its efficacy [106]. Propolis-loaded cryogels, incorporating polyvinyl alcohol (PVA) and polyacrylic acid, exhibited material properties and antibacterial effects suitable for bactericidal dressings.

### 3.4. Acrylate- and Methacrylate-Based Cryogels

Cryogels composed of acrylate and methacrylate polymers have emerged as promising materials in drug delivery and biomedical engineering. Extensive studies and research proposals have been conducted to investigate their diverse applications. One proposal suggests the use of biodegradable and thermoresponsive cryogels containing simvastatin for controlled drug release in bone tissue engineering [107]. Another proposal introduces a hybrid system of pHEMA and halloysite nanotubes (HNTs) embedded with thymol, providing sustained drug delivery for wound healing, particularly in space missions [25]. Cryogels have also demonstrated efficacy as chromatographic support for plasmid purification, meeting FDA standards [108]. Furthermore, cryogels can be developed as imprinted discs for the controlled release of 5-fluorouracil or as implantable drug delivery systems for cancer treatment [109,110]. These studies underscore the potential of cryogels in creating customized drug delivery systems and advancing biomedical applications.

Apart from drug delivery, cryogels have shown promise in environmental applications. Polymeric cryogels have proven effective in removing emerging contaminants from water, highlighting their impressive adsorption capabilities for a wide range of pollutants [111]. Additionally, a temperature-responsive cryogel composed of poly(hydroxyethyl acrylate-co-phenyl vinyl sulfide) has been investigated for delivering doxorubicin to tumor environments, utilizing temperature, oxidation, and NIR radiation-induced changes to control drug release [112]. Cryogels have also been utilized for the controlled release of verapamil hydrochloride, demonstrating their macroporous structure and sustained release properties at physiological temperatures [113]. Finally, the scaling laws and thermoresponsive behavior of charge-balanced terpolymer hydrogels and cryogels have been explored, providing valuable insights for designing more efficient drug delivery systems [114]. These studies highlight the versatility and adaptability of cryogels in tailoring drug delivery and addressing environmental challenges.

### 3.5. Acrylamide-Based Cryogels

The application of acrylamide cryogels has been extensively explored in various fields. One study [115] focuses on the fabrication of conductive cryogels by incorporating polyaniline or polypyrrole nanoparticles. These hybrid cryogels exhibit desirable properties such as conductivity, photothermal response, and pressure-dependent conductivity. They show potential for use in electrical devices, tissue engineering scaffolds, drug delivery vehicles, and electronic skin. Additionally, these cryogels demonstrate temperature and near-infrared (NIR) light-sensitive on–off switches, as well as water-triggered shape memory on–off switches.

Another study [116] investigates macroporous poly(acrylamide/methacrylic acid) (PAM) cryogels for drug adsorption. These cryogels have high macroporosity and swelling ability. The study highlights their efficient adsorption of clarithromycin through a combination of swelling and diffusion-controlled mechanisms. This suggests their potential use in drug delivery, particularly in controlled release systems.

Researchers have proposed the use of hydrogel phantoms with varying porosity and stiffness to enhance the prediction of in vivo performance for in situ forming implants (ISFIs) used in sustained drug delivery [117]. The study suggests that macroporous phantoms with a stiffness of 30 kPa better mimic the burst release observed in vivo. By investigating the relationship between phantom properties, implant microstructure, and drug release, hydrogel phantoms show promise in improving the correlation between in vitro and in vivo performance of ISFIs, facilitating their clinical use.

Researchers have also reported the development of macroporous cryogel scaffolds made of N-(2-hydroxypropyl) methacrylamide (HPMA) modified with a peptide for biomimetic adhesion sites [118]. These cryogels possess suitable mechanical properties and are non-toxic to human adipose-derived stem cells. The modified cryogels, enriched with the peptide, support cell attachment, spreading, and proliferation, making them promising candidates for various stem cell applications.

### 3.6. Other Synthetic Cryogels

Zwitterionic cryogels have attracted considerable attention for their diverse applications, as evidenced by a range of research proposals and studies. One study [119] explores their potential as a delivery system for chemoimmunotherapy in cancer treatment. The cryogels demonstrate precise and continuous release of chemotherapeutic drugs and immune adjuvants near tumors, leading to improved therapeutic outcomes. Additionally, the cryogels support cell infiltration, making them a promising platform for vaccines.

In another study [120], zwitterionic cryogels are investigated for sustained protein release. The study highlights their high loading efficiency and provides insights into their superior protein loading and release properties. Furthermore, research [121] has suggested the use of zwitterionic cryogels loaded with cerium oxide nanoparticles and microRNA146a for the treatment of chronic wounds in diabetic patients. These cryogels exhibit unique physical properties and sustained release mechanisms that promote wound healing.

Turning to neuroprosthetics, ref. [122] presents the development of conducting polymer-entrapped cryogels. These cryogels offer a stable and long-term electronic-tissue interface and hold potential for therapeutic delivery. Moreover, ref. [123] proposes a strategy to enhance the bioavailability and stability of curcumin using cryogelation to prepare composite cryogels. This approach effectively modulates the hydrophobicity of the cryogel carrier, resulting in enhanced curcumin release.

Additionally, other notable studies are found in the field. One study [124] focuses on the development of monosaccharide-responsive hydrogel and cryogel scaffolds, capable of transforming between gel and sol phases. These scaffolds show promise for cell sheet engineering and analyte-responsive drug delivery. Another research proposal [125] explores the synthesis of magnetic field-responsive p(VPA) hydrogel nanoparticles, with potential applications in drug delivery and environmental absorbents.

Furthermore, another proposal [126] describes the synthesis of macroporous ionic composite cryogels, serving as carriers and chromatographic materials. These cryogels demonstrate high reusability and selectivity in separation processes. In a related context, another study [127] investigates the porosity and sorption properties of ionic interpenetrating polymer network composite cryogels, shedding light on their potential as drug carriers. Lastly, another research proposal [128] focuses on fabricating fast-responsive IPN hydrogels with tunable properties, holding promise for controlled drug release.

### 3.7. Other Cryogel Materials

The potential of cryogels in drug delivery has been explored through various studies and research proposals. One study [129] proposed a calcium phosphate-based cryogel as a biomaterial for drug delivery, demonstrating the preservation of alkaline phosphatase (ALP) enzyme activity. Another study [130] focused on the development of low-density organic–inorganic hybrid aerogels with unique properties suitable for stimuli-responsive materials and drug delivery systems. Furthermore, researchers proposed the use of cryogel scaffolds to enhance the stability and control of antimicrobial peptides in hydrogel scaffolds, enabling specific loading and controlled release capabilities [131]. Additionally, another study presented the development of photosensitive polymeric nanoparticles for the recognition and determination of ubiquitin, offering potential applications in biosensors and drug delivery systems [132].

In the field of tissue engineering, cryogels have shown promise as scaffolds for tissue regeneration. One study developed a Kefiran-based scaffold using freeze gelation for drug delivery and tissue engineering applications, demonstrating high porosity, sustained release properties, and support for cell metabolic activity [133]. Magnetic microcryogels were proposed for the formation and manipulation of microtissues, showcasing their potential for in vitro tissue engineering, drug testing, and cell therapy applications [134]. Additionally, a composite cryogel scaffold was developed for local antibiotic delivery to treat bone and joint infections, exhibiting biocompatibility, osteogenic capacity, and anti-infective characteristics [135]. Silk-based cryogel scaffolds were also explored, discussing fabrication strategies and the tunability of silk-based biomaterial properties to address challenges in various bioengineering disciplines [136].

In the field of dermatology, cryogels have been investigated for their potential in drug delivery and the treatment of skin diseases. Super-macroporous cryogels from non-modified high-molar-mass dextran were proposed as drug carriers, evaluating their crosslinking efficacy and physico-mechanical properties [137]. Macroporous cryogels were developed for the topical delivery of curcumin in the treatment of cutaneous T-cell lymphoma (CTCL), demonstrating sustained release and comparable cytotoxicity to the free drug [138]. Hyaluronic acid-based shape-memory cryogels were developed as minimally invasive biomaterials for cartilage defect repair, showing enhanced chondrocyte viability and matrix biosynthesis compared to hydrogels [139]. The potential applications of hyaluronic acid in inflammatory skin diseases were also explored, highlighting its use in drug delivery systems for enhanced penetration and localized release of anti-psoriasis drugs and inflammation inhibition in atopic dermatitis [140].

### 3.8. Developments

The research pattern presented in Table 1 provides insight into the extensive exploration of cryogel materials in pharmaceutical and biomedical applications. It is evident that cryogels have emerged as versatile platforms with immense potential, particularly in the fields of drug delivery and tissue engineering. The volume of research conducted on various hydrogel systems, specifically cryogels, underscores their significant role in addressing a wide range of diseases and therapeutic applications.

Alginate-based cryogels have been studied extensively for their applications in drug delivery and tissue engineering. These cryogels have demonstrated their versatility as platforms for the controlled release of therapeutic agents [26,27,28,30,31,32]. Similarly, cryogels based on chitosan, another polysaccharide, have shown promise in multiple applications, including controlled drug delivery, peripheral nerve regeneration, bone regeneration, and wound healing [21,33,34,38,39,40,41,42,43,44,49,50,52,53].

Gelatin cryogels have also received significant attention, with research focusing on their applications in osteomyelitis, osteoporosis, damaged tissue therapy, corneal keratocyte growth, vascular endothelial growth factor (VEGF) delivery, and transdermal drug delivery [54,55,56,58,59,60,61,62,63,64,66,67,68,141].

Polysaccharides and their hybrids, such as agarose, carrageenan, and cellulose-based cryogels, have shown potential as drug delivery systems and scaffolds for tissue engineering. Their applications range from cartilage regeneration to wound healing and controlled release [22,75,76,77,78,79,80,81,82,83,84].

Cryogels based on PEG derivatives offer unique advantages in immunotherapeutic treatments, slow-releasing drug reservoirs, nerve growth promotion, and precision delivery of signaling proteins [23,24,87,88,142]. Additionally, cryogels incorporating PVOH (polyvinyl alcohol) and PVOH hybrids have demonstrated controlled drug release, wound healing properties, and applications in orthopedic and cardiovascular devices [90,91,92,93,94,95,96,97,98,143,144,145,146,147,148,149].

Furthermore, cryogels based on acrylate/methacrylate polymers have been extensively studied for controlled drug release, incorporating specific drugs and substances. Other synthetic cryogels have also shown diverse applications in drug delivery, tissue engineering, and controlled release [25,107,109,110,114,150].

**Table 1:** Research pattern for cryogel materials used in pharmaceutical and biomedical applications.

Alginate-Based Cryogel		
Material Used	Main Outcomes	Ref.
Alginate cryogel loaded with chemoimmunotherapy drug-loaded nanoparticles Sp-AcDEX NPs and Nutlin-3a	Chemoimmunotherapy drug delivery system for cancer treatment, enhancing accumulation of Sp-AcDEX NPs in tumor tissue and inducing immunogenic cell death with Nutlin-3a	[26]
Macroporous alginate cryogel incorporated with gold nanorods (GNRs)	Controlled drug delivery system for Mitoxantrone with on-demand release through near-infrared irradiation, suppressing tumor growth in vivo	[27,28]
Nanocomposite hydrogel formed by bio-orthogonal crosslinking of alginate using tetrazine-norbornene coupling and pre-adsorbed charged Laponite nanoparticles	Sustained, bioactive release of therapeutic proteins with precise tuning of release kinetics, simplifying the design of hydrogel drug delivery systems	[27,28]
Sodium alginate-based aerogel with photosensitizers and phenylboronic acid	Antibacterial photodynamic wound dressing with improved solubility, hemostasis capacity, and antibacterial activity against *Staphylococcus aureus*	[29]
Alginate and carboxymethyl-cellulose-based cryogel neural scaffold	Injectable surgical scaffold for minimally invasive delivery of an extended living neuronal network, with high local Young’s modulus for neuronal network protection and soft macroscopic scale for easy injection	[30,31,32]
3D porous scaffold made from carrageenan and alginate with EDC/NHS cross-linker	Scaffold with a porous and interconnected structure, good physical and mechanical stability, higher cell attachment, better cellular response, and higher metabolic activity than matrices with other cross-linkers	[31,32]
Autoclavable cryogels made from several naturally derived polymeric precursors (alginate, hyaluronic acid, and gelatin)	Maintain their structural and physical properties, including their syringe injectability signature, after autoclave sterilization	[31,32]
**Chitosan-Based Cryogel**		
**Material Used**	**Main Outcomes**	**Ref.**
Hydrophobically modified chitosan cryogel	Potential material for controlled drug delivery applications	[33]
Chitosan aerogel microparticles	Suitable for pulmonary drug delivery systems; effects of chitosan molecular weight, polymer concentration, and tripolyphosphate concentration on drug release were investigated	[34]
Chitosan sponges Cross-linked with glutaraldehyde	It possesses antibacterial, antioxidant, and controlled delivery properties for plant-derived polyphenols, showed remarkable shape recovery, good radical scavenging activity, and strong antibacterial properties against both Gram-positive and Gram-negative strains	[35]
Cryogel–microparticle composite with polymeric network and microstructured biocompatible pore surface	Incorporating microparticles (MPs) into the polymeric network of cryogels to deliver bioactive molecules, demonstrated good biocompatibility with the growth of various cell lines, potential for delivering bioactive/drug molecules to cells growing in 3D conditions	[36]
Double cryogel system, gelatin/chitosan cryogel (GC) surrounded by Gelatin/heparin cryogel (GH)	The double cryogel system (DC) is used for dual drug delivery with different release kinetics to induce bone regeneration. The GH layer loaded with VEGF induces initial release, while the GC layer loaded with BMP-4 induces sustained release.	[37]
Chitosan and nanofibrillated cellulose hydrogel	Investigated as a biomaterial membrane for peripheral nerve regeneration due to its mechanical strength, thermal resistance, slow rate of swelling, and non-toxicity to Schwann cells. Used in vivo with autologous implant to promote functional recovery within 15 days.	[38]
Chitosan-clinoptilolite 3D biocomposites	Investigated for their potential as drug carriers. Biocomposites with higher content of clinoptilolite were found to have a more ordered porous structure and lower water uptake. Effective drug release was observed in phosphate buffer saline, suggesting potential for drug delivery systems.	[39]
Pectin/Chitosan Composite Cryogel	Potential candidate for biomedical applications due to improved mechanical strength, surface morphology, degradation time, adhesion to biological tissues, and biocompatibility.	[40]
Poly(N-isopropylacrylamide) scaffold loaded with chitosan/bemiparin nanoparticles	Used for tissue engineering and controlled release of heparin. Cryogel exhibits a highly porous structure, is non-cytotoxic, and exhibits excellent properties for application in tissue engineering.	[41]
Chitosan/Hydroxyapatite/Heparin/PVA Composite Cryogel Scaffold	Potential for bone regeneration and tissue engineering applications, as it efficiently immobilizes BMP-2 and supports differentiation of bone marrow mesenchymal stem cells into the osteogenic lineage.	[42,43]
Thiolated Chitosan/Oxidized Dextran/Locust Bean Gum Semi-IPN Cryogel	Promising candidate for use as a hemostatic dressing due to its improved mechanical strength, decreased hydrophobicity, increased swelling ratio, and enhanced hemostatic properties. Cryogel also exhibits good cytocompatibility, blood compatibility, and hemostatic potential.	[42,43]
Chitosan-graft-poly(N-isopropyl acrylamide)/PVA	Development of a smart polymeric vehicle for antifungal drug delivery for mucosal applications and various parameters of cryogels were also characterized.	[44]
Chitosan/2-hydroxyethylcellulose cryogel	Development of a pH-sensitive cryogel for drug delivery applications. The cryogels were characterized for physico-mechanical properties and bioadhesive properties.	[45]
Methacrylated chitosan/chondroitin sulfate cryogel microparticle (CMP) system	Development of an injectable CMP system for growth factor delivery in tissue regeneration applications. The cryogel microparticles were characterized for in vitro and in vivo neovascularization using rhVEGF.	[46]
Chitosan-based polyelectrolyte complex cryogels	Development of a sustainable strategy to fabricate drug delivery systems using polyelectrolyte complex cryogels physically stabilized by spontaneous interactions. The cryogels were characterized for their structure, morphology, and drug release properties.	[47]
Glutaraldehyde-crosslinked chitosan cryogels	Fabricated with superamphiphilicity and high separation efficiency for various surfactant-stabilized oil-in-water emulsions under continuous flow mode, make them promising for applications in separation processes driven by gravity or a peristaltic pump.	[151]
Colloidal chitosan/k-carrageenan/carboxymethylcellulose sodium salt cryogel	Encapsulation of curcumin in a controlled release system.	[152]
Chitosan/xanthan gum polyelectrolyte complex (PEC) aerogel	Development of bone-like structured aerogels suitable for biomedical and environmental applications.	[48]
Chitosan/citric acid/silver nanoparticle cryogel	Development of a shape memory cryogel dressing for skin wounds, promoting hemostasis, blood cell adhesion, and wound healing.	[49,50]
Chitosan and gluconic acid conjugate cryogels	Development of physically crosslinked chitosan cryogels as practical wound dressings, which retained the same biological properties as the pre-autoclaved ones and showed enhanced resistance to enzymatic degradation.	[49,50]
Methacrylic acid/acrylamide or 2-hydroxyethyl methacrylate cryogel 1st network crosslinked with chitosan/poly(ethyleneglycol) diglycidyl ether 2nd network	Used for designing macroporous hydrogels for controlled release of macromolecular drugs	[51]
Chitosan/silk fibroin/tannic acid/ferric ion cryogel	Developed as a wound dressing with stimuli-responsive photothermal therapy and good antibacterial and cell-proliferative properties	[49,50,52]
Chitosan-gelatin-chondroitin sulfate/nano-hydroxyapatite-gelatin cryogel scaffold	Used for tissue-engineering repair of articular cartilage injuries with potential therapeutic approaches for osteochondral repair	[21]
Chitosan-gelatin-polypyrrole 3D scaffolds with chitosan and gelatin microspheres	Evaluated for delivering alpha-ketoglutarate (alpha-KG) to cells in tissue engineering applications	[53]
**Gelatin-Based Cryogel**		
**Material Used**	**Main Outcomes**	**Ref.**
Gelatin-based cryogel system embedded with CaCO_3_ microspheres and ciprofloxacin hydrochloride	Address the condition of osteomyelitis and osteoporosis, sustained drug release for up to 21 days, significant increase in cell viability and alkaline phosphatase levels in rat osteoblasts	[54]
Injectable 3D microscale cellular niches using biodegradable gelatin microcryogels	Optimize cell therapy for damaged tissues or organs, facilitate cell protection during injection and in vivo cell retention, survival, and therapeutic functions, superior therapeutic efficacy for treating critical limb ischemia in mouse models	[59]
Gelatin microcryogels loaded with hASCs	Injectable gelatin microcryogels were loaded and primed with human adipose-derived stem cells to create 3D cellular micro-niches that accelerate wound healing.	[60]
Injectable cryogels made of gelatin methacryloyl and poly(ethylene)glycol	Tunable degradability and porosity suitable for cell and drug delivery applications.	[56]
Cryogel scaffold composed of gelatin	Evaluation of polyelectrolyte multilayer microcapsules for controlled release of transforming growth factor-beta 1 (TGF-beta 1) in gelatin-based hydrogels and cryogel scaffolds for tissue engineering applications.	[57]
Gelatin-based cryogel with cellulose nanocrystal (CNC) and poly-amidoamine (PAMAM) dendrimer	For sustained drug release and improved mechanical properties.	[58]
Cryogenic Gelatin-Hyaluronic Acid Composite	Elastic scaffold for 3D bioprinting with continuous interconnected macroporous structure, allowing for cell attachment, viability, and proliferation	[61]
Nanocellulose and Gelatin Composite Cryogel	Controlled drug release carrier with controllable and sustained release of drug 5-FU	[62]
Nanosilicate and gelatin methacrylate Cryogel	Tough, macroporous hydrogel for drug delivery with sustained release under physiological conditions, reducing endothelial cell injury caused by nutrient deprivation	[63]
Gelatin/ascorbic acid (AA) cryogels	Carriers for corneal keratocyte growth in corneal stromal tissue engineering	[64]
Gelatin/heparin cryogel and BMP-2-loaded cryoelectrospun poly(epsilon-caprolactone) hybrid scaffold	Bone regeneration scaffold with enhanced mechanical support and sustained release of BMP-2	[66]
Gelatin and heparin-based injectable cryogel	Carrier for in vivo cell and growth factor delivery in hindlimb ischemic disease	[67]
Amino acid-based functional monomer with HEMA and gelatin	Development of degradable molecularly imprinted cryogel for pH-responsive delivery of doxorubicin	[65]
Usnic acid encapsulated in Rhamnolipid biosurfactant nanoparticles enriched cryogel nanocomposite scaffold	Design of cryogel nanocomposite scaffold with dual properties of bone regeneration and antibacterial effect for the treatment of osteomyelitis	[55]
Macroporous composite biomaterial of gelatin-hydroxyapatite-calcium sulphate	Development of biomaterial with spatio-temporal delivery of rhBMP-2 and ZA leading to increased bone formation compared to commercially available carriers	[68]
Gelatin-based cryogel	Use of gene delivery and tissue-engineering approaches for regenerating cartilage tissue lost due to trauma, tumor surgery, or congenital defects	[69]
**Polysaccharides and Their Hybrids-Based Cryogel**		
**Material Used**	**Main Outcomes**	**Ref.**
Hydrophobically modified agarose cryogels (HMA cryogels)	Drug delivery systems, low cytotoxicity, adsorb more hydrophobic dye, and controlled release of dye	[75]
Mesophyll-inspired agarose cryogel (MAC)	Component of leaf-inspired micropump (LIM) for smart microfluidic applications, adjusts delivery rate of therapeutic liquid in response to temperature changes	[76]
Agarose-based cryogels, including cellulose fibers and microparticles	Water absorption and retention ability with high structural stability, potential for drug delivery devices	[77,78]
Carrageenan cryogels loaded with alpha-aminophosphonates	Improved mechanical properties and sustainable release of antimicrobial compounds for potential use against *S. aureus*	[79]
Kappa-Carrageenan (kappaC) matrix	Transdermal controlled delivery patch for Metformin, with enhanced drug release-permeation and diffusion coefficients when an electrical potential is applied	[80]
LBG, XG, and MG-based cryogel scaffolds	Promising properties for cartilage and soft tissue engineering and drug delivery, sustained release of Kartogenin achieved	[81]
Cellulose cryogels microsphere	For wound healing, tissue regeneration, and drug delivery	[22,82]
CNF-PNIPAm hybrid	Synthesized temperature-sensitive polymer-modified cellulose nanofibril (CNF) cryogel microspheres with potential application in controlled drug release.	[83]
Biopolymer cryogels	Developed as an effective delivery system for curcumin in the form of Curcumin-nanostructured lipid carrier-loaded oleogels (Cur-NLC-OGs). Biopolymer cryogels were used to stabilize and self-stand the Cur-OGs.	[84]
Super-macroporous hydroxypropyl cellulose (HPC) cryogels embedded with stabilized core-shell micelles (SPM)	For sustained delivery of poorly water-soluble drugs	[153]
Hydroxyethyl cellulose (HEC) cryogels with polymeric micelles	Sustained topical delivery of hydrophobic natural substances such as cannabidiol	[85]
Composite cryogels (PVP/NaCMC) microspheres loaded with Mupirocin (MP)	Controlled release of antibiotic Mupirocin	[86]
Enzymatically modified starch cryogels	Carriers of active molecules for drug delivery systems and potential applications in biomedical and food packaging scenarios	[154]
Composite cryogels (OPS or OWS with DMAEM) fabrication of semi-IPN cryogels using N,N-dimethylaminoethyl methacrylate (DMAEM) and oxidized starches oxidized potato starch (OPS) or oxidized wheat starch (OWS)	For controlled drug release in simulated gastric fluid at pH 1.3	[155]
**PEG Derivative-Based Cryogel**		
**Material Used**	**Main Outcomes**	**Ref.**
Poly(ethylene glycol)-heparin cryogel scaffold	Promotes proliferation and survival of bsAb-releasing-MSCs, constant release of sustained and detectable levels of bsAb, and effective in triggering T-cell-mediated anti-tumor responses for treatment of acute myeloid leukemia (AML)	[87]
Activated carbonate group- with PEG functionalized cryogels	Slow-releasing drug reservoirs for anticancer drug delivery, with 7-fold higher drug release compared to hydrogels	[23]
Poly(ethylene glycol) diacrylate and maleimide-functionalized heparin cryogels	Sustained delivery of growth factors for tissue engineering and regenerative medicine applications, with the ability to load and release nerve growth factor over a period of 2 weeks and induce neurite outgrowth	[88]
Dextran methacrylate and polyethylene glycol dimethacrylate cryogel	Spongy scaffold for promoting the delivery of biomolecules in drug delivery and tissue engineering applications, with improved swelling, increased interconnected porosity, and higher mechanical resistance than conventional hydrogels	[142]
StarPEG-heparin cryogel	Allows for tunable, long-term delivery of different signaling proteins for tissue engineering applications, inducing local differences in protein concentration, and inducing neuronal differentiation of cells	[24]
Dendrimer cryogel made of hyperbranched amine-terminated polyamidoamine (PAMAM) dendrimer G4.0 and linear polyethylene glycol (PEG) diacrylate	Superelastic network with high compression elasticity, super resilience, and stability at acidic pH, suitable for biomedical applications due to its self-triggered degradation at physiological pH	[156]
Poly(ethylene glycol) diacrylate microcryogels	Utilized in a syringe-based 3D culture system for the mechanical preconditioning of mesenchymal stromal/stem cells toward nucleus pulposus (NP)-like cells, with potential for NP regeneration	[89]
**PVOH-Based Cryogel**		
**Material Used**	**Main Outcomes**	**Ref.**
PVA/ZnO/FA nanocomposite	Drug delivery application with controlled release of fulvic acid	[90]
PVA cryogel with thermochromic ink	Thermochromic material for temperature estimation in ultrasound therapy	[91]
PVA cryogel	Enhancing solubility of Simvastatin for use as a prolonged-release cryogel matrix for hydrophobic drugs and potential biomaterial for tissue engineering	[92,143]
Poly(vinyl) alcohol and propylene glycol cryogel	Used as a hydrophilic active wound dressing loaded with trans-resveratrol for controlled release and reduced irritation	[144]
Composite cryogels containing PVA particle and porous adsorbent particles	Used for capturing glycoproteins and evaluated for repeated use in batch and chromatographic experiments	[145]
Polyvinyl alcohol cryogel with high-methoxylated pectin	Used for the controlled release of the antibiotic enrofloxacin, and a two-layer film system was designed to modulate the release rate of the drug	[146]
Polyvinyl alcohol cryogel with urea additives	Used as a polymeric carrier in drug delivery systems with widened macropores, and the release rate of the drug depends on the urea content in the initial PVA solution	[147]
Highly porous composite PVA cryogels loaded with drug molecule	Highly porous composite cryogels loaded with PHB microbeads containing simvastatin for controlled drug delivery	[93]
PVA/iron oxide NPs	Cryogels for thermally triggered drug release based on shape-selective heat transfer using magnetic nanoparticles coated with acetaminophen	[148]
Polyvinyl alcohol (PVA)	Investigation of PVA cryogel for iontophoretic transdermal drug delivery	[94,149]
PVA and its nanocomposites	Used for biomedical and medical device applications due to its unique mechanical properties that can be tailored to match soft tissues	[96]
PVA-NLC, nanostructured lipid carriers loaded with drug molecules	Serves as an adhesive film that contains nanostructured lipid carriers loaded with olanzapine and simvastatin for transdermal treatment of psychiatric disorders	[95]
PVA-DNA gel matrices	Examines factors that affect the release rate of deoxyribonucleic acid (DNA) from PVA hydrogels and its blend with DNA. Investigates the potential of designing controlled DNA release PVA-based devices	[97,98]
**PVOH Derivatives-Based Cryogel**		
**Material Used**	**Main Outcomes**	**Ref.**
Molecularly imprinted cryogel	Used for solid-phase extraction of propranolol from aqueous solution and complex plasma sample due to its high selectivity and stability	[99]
PVA and gelatin cryogels	Evaluated for their water-uptake potential, influence of various factors on water sorption, and biocompatibility for potential use in biomedical applications	[100]
PVA and egg albumin cryogels	Investigated for their water sorption capacity, swelling behavior, in vitro biocompatibility, thermal and morphological characterization for potential use in the biomedical field	[101]
pH-sensitive hydrogel made from a blend of polyvinyl-alcohol, polyacrylic acid, and synthetic hydroxyapatite	Explored for specific drug delivery applications due to their smart properties. Investigated the effects of hydroxyapatite on mechanical strength, bioactivity, and drug release profiles, with promising results seen in terms of swelling, gel fraction, and drug delivery	[102]
Gum tragacanth-polyvinyl alcohol (GT-PVA) cryo- and xerogels	Prepared and evaluated for their physical, mechanical, and release properties, including the effect of GT ratio and inclusion of silymarin. Found to have potential applications due to their porosity, microstructure, and mucoadhesive properties	[157]
poly(vinyl alcohol),pullulan and zeolite Composite cryogel	Controlled release of Enalapril Maleate drug used to treat hypertension and heart failure	[104]
Dual network hydrogel of poly(vinyl alcohol) cryogel and sodium alginate	Synthesized for potential biomedical applications. Can absorb and retain fluids, incorporate biological molecules/drugs	[105]
PVA and polyacrylic acid Cryogel carrier system	Controls the release rate of extracted propolis for enhanced efficacy. Can be used as bactericidal dressing	[106]
Two-phase hydrogel prepared by physically imbedding a xerogel in the core of a cryogel that was freeze thawed with PVA and PAA	Temperature-sensitive drug delivery systems, drug release at a slower rate from hydrogels containing acrylic acid	[158]
PVA/HA pH-responsive cryogel	Drug delivery system for the treatment of psoriasis, significant decrease in toxicity, swelling and drug release at pH 5.5	[103]
**Acrylate and Methacrylate Base Cryogel**		
**Material Used**	**Main Outcomes**	**Ref.**
NIPA and HEMA-lactate-Dextran-based biodegradable and thermoresponsive cryogels	Potential use in bone tissue engineering with controlled drug release capabilities	[107]
Poly 2-hydroxyethyl methacrylate (pHEMA) and halloysite nanotubes (HNTs) embedded with thymol (Thy)	Sustained release drug delivery system for wound healing in space	[25]
Poly(2-hydroxyethyl methacrylate)	Purification of plasmid expressing Influenza hemagglutinin gene	[108]
Metal-chelate monomer N-methacryloyl-L-histidine, hydroxyethyl methacrylate and Cu(2+) ion	Imprinted cryogel discs for delivering chemotherapy drug 5-fluorouracil (5-FU)	[109]
Cryogel-based molecularly imprinted membranes	Implantable drug delivery system for controlled release of antineoplastic agent Mitomycin C for cancer treatment	[110]
HEMA-based cryogels	Potential candidates for controlled drug delivery systems in biomedical applications	[150]
Polymeric cryogels	Efficient removal of emerging contaminants from water and suitable for drug delivery applications	[111]
Poly(hydroxyethyl acrylate-co-phenyl vinyl sulfide) cryogel	Suitable anticancer drug delivery carrier	[112]
PETEGA cryogels	Controlled drug release for verapamil hydrochloride	[113]
Terpolymer hydrogels and cryogels	Potential to improve loading capacity of polymers used in anticancer drug delivery systems	[114]
**Other Cryogels**		
**Material Used**	**Main Outcomes**	**Ref.**
Poly(N-isopropylacrylamide) cryogels with conducting polyaniline or polypyrrole nanoparticles	Potential application in electrical devices, tissue engineering scaffolds, drug delivery vehicle, and electronic skin	[115]
Zwitterionic cryogels	For drug delivery, chemoimmunotherapy, and long-term release of proteins	[119,120,121]
Calcium phosphate-based cryogel encapsulating alkaline phosphatase (ALP)	Preserves activity of ALP and has chemical and structural properties determined using X-ray diffraction, helium pycnometry and mercury porosimetry	[129]
Furan-based cryogel (CG) scaffold covalently conjugated with maleimide-modified antimicrobial peptides	Rapid loading and release of therapeutic peptides with high water uptake; “on-demand” photothermal heating upon NIR irradiation	[131]
Hybrid and composite biomaterials	To enhance mechanical strength and porosity	[126,130,135]

## 4. Challenges and Perspectives

Cryogels have emerged as highly versatile and promising biomaterials with numerous applications in various biomedical fields. Extensive research has been conducted to explore the potential of cryogels composed of different materials, such as alginate, chitosan, silk fibroin, gelatin, agarose, and cellulose derivatives, for tissue engineering, controlled drug delivery, regenerative medicine, and other therapeutic strategies [28,29,30,31,32,33,34,35,36,37,38,39,40,41,42,43,44,45,46,47,48,49,50,51].

One significant area of focus is alginate-based cryogels, which hold promise in cancer therapy [26]. Optimization of protein release kinetics in alginate-based nanocomposite hydrogels also needs to be addressed [27,28], along with a thorough evaluation of the clinical efficacy and long-term effects of antibacterial photodynamic wound dressings composed of sodium alginate and photosensitizers [29]. Injectable neural scaffolds made from alginate and carboxymethyl-cellulose show promise in neural tissue engineering but require further validation and research [30,31,32]. Careful selection of cross-linkers is necessary for optimal physical and mechanical stability of 3D porous scaffolds made from carrageenan and alginate [31,32]. Additionally, studying the long-term effects of sterilization on polymeric cryogels composed of alginate, hyaluronic acid, and gelatin is crucial [31,32].

Chitosan-based cryogels also demonstrate significant potential in various biomedical applications. Hydrophobically modified chitosan cryogels exhibit optimized strength and potential as biomedical materials [33]. Chitosan aerogel microparticles prepared using supercritical fluid technology are suitable for pulmonary drug delivery systems [34]. Chitosan sponges possess antibacterial, antioxidant, and controlled delivery properties for plant-derived polyphenols [35]. Cryogel–microparticle composites have potential for delivering bioactive/drug molecules to cells in 3D conditions [36]. A double cryogel system for dual drug delivery demonstrates enhanced bone regeneration [37]. Chitosan and nanofibrillated cellulose biomaterial membranes show ideal nerve regeneration potential [38]. Chitosan-clinoptilolite 3D biocomposites act as effective drug carriers with controlled and sustained release profiles [39]. Composite cryogels based on pectin and chitosan exhibit improved properties for biomedical applications [40]. PolyNIPAM thermosensitive macroporous scaffolds loaded with chitosan/bemiparin nanoparticles demonstrate excellent properties for tissue engineering and controlled release of heparin [41]. Chitosan-based cryogel scaffolds show potential for bone regeneration and tissue engineering [42,43]. Chitosan-based wound dressings with enhanced mechanical strength, decreased hydrophobicity, increased swelling ratio, and hemostatic properties show promise [42,43]. Physically crosslinked gel formulations of chitosan-graft-poly(N-isopropyl acrylamide) and polyvinyl alcohol hold potential as smart polymeric vehicles for topical drug delivery [44]. pH-sensitive chitosan-based cryogels offer a promising approach for controlled and targeted drug delivery [45]. Injectable cryogel microparticles enable sustained release of growth factors for tissue regeneration [46]. Chitosan-based polyelectrolyte complex (PEC) cryogels and aerogels show sustainable release and thermal stability for effective drug delivery [47,48]. Low-cost shape memory cryogel dressings composed of chitosan and citric acid hold promise for skin wound healing [49,50]. Physically crosslinked chitosan cryogels demonstrate resistance to enzymatic degradation and potential as practical wound dressings [49,50]. Macroporous hydrogels designed using cryogelation and interpenetrating polymer networks (IPNs) show potential for controlled release of macromolecular drugs [51].

Furthermore, cryogels composed of various materials have been investigated as scaffolds or carriers for therapeutic agents, and their properties have been optimized to achieve controlled and sustained drug release profiles [21,33,34,35,36,37,38,39,40,41,42,62,63,70,71,73,74]. The incorporation of bioactive molecules within cryogel matrices has also shown promising results for enhanced therapeutic efficacy [21,55,57,59,60,65,66,67,68,69].

Moreover, cryogels have been explored for gene delivery, cartilage tissue regeneration, and as drug delivery systems for cancer treatment and water purification [69,109,110,111]. They have demonstrated characteristics such as improved encapsulation efficiency, sustained release profiles, enhanced drug solubility, and controlled release behavior, making them suitable for a wide range of therapeutic applications [22,23,24,25,84,85,86,87,88,90,92,93,94,95,97,98,99,100,101,102,103,104,105,106,107].

Despite these advancements, there are still challenges that need to be addressed. Optimization of cryogel properties for specific applications, improvement of release profiles, enhancement of mechanical strength, and achievement of long-term stability are areas that require further research [22,23,24,25].

However, the studies conducted on cryogels have provided valuable insights into their limitations, challenges, and future potential, paving the way for the development of innovative and effective biomaterials for biomedical applications.

Moving forward, several key research directions can further advance the field of cryogels:

Integration of Advanced Manufacturing Techniques: Future research could concentrate on integrating cutting-edge manufacturing methods like 3D printing or electrospinning to advance the field of cryogels [21]. These methods have the potential to improve the control over the structure and characteristics of cryogels, enabling the creation of biomaterials that are specifically tailored and perform better.

Biomimetic Cryogel Design: Investigating the creation of cryogels that resemble particular tissue architectures or the extracellular matrix may provide new opportunities for tissue engineering and regenerative medicine [22]. Cryogels may be able to support cell growth, differentiation, and general tissue regeneration more effectively by incorporating cues from natural tissues.

Long-Term Stability and Biodegradability: Future studies should focus on enhancing cryogels’ long-term stability and biodegradability, particularly for uses where controlled degradation is preferred [23]. For cryogels to be used in various biomedical applications, tunable degradation rates and an understanding of their biocompatibility profiles are essential.

Combination Therapies and Drug Delivery Systems: The field could be significantly advanced by further research into the use of cryogels as controlled drug delivery systems or carriers for combination therapies [24]. It may be possible to enhance therapeutic outcomes and lessen side effects by incorporating multiple therapeutic agents into cryogel matrices or creating cryogels with controlled release properties.

In Vivo Evaluation and Clinical Trials: To ascertain the safety and efficacy of cryogels as they are developed, more emphasis should be placed on in vivo testing and preclinical research [25]. It would be necessary to conduct carefully planned animal studies and eventually move toward clinical trials in order to translate cryogel-based therapies into clinical practice. Literature shows one study on alveolar ridge preservation that used a gelatin cryogel scaffold with small-sized and regular-sized organic bovine bone particles. The results showed that the small-sized particles filled the socket, reduced ridge atrophy, and provided similar ridge stability as the regular-sized particles [159]. Currently, a Phase II clinical trial is ongoing, investigating the use of an erythropoietin/isosorbide dinitrate loaded cryogel scaffold for treating diabetic foot ulcers [160].

Additionally, there are several practical challenges that need to be addressed for the successful implementation of cryogels in real-world applications. Scaling up the production process while maintaining desired characteristics and performance of cryogels is crucial for their practical use [21]. Ensuring reproducibility, consistency from batch to batch, and developing cost-effective manufacturing techniques are important considerations.

Moreover, maintaining the structural integrity and mechanical stability of cryogels in the face of real-world conditions such as mechanical stress, temperature variations, and humidity fluctuations is a significant challenge [25]. Enhancing the long-term stability and robustness of cryogels is essential for their practical applications.

Mass transfer constraints, particularly for large molecules or densely packed cryogel structures, can impede the transport of fluids and solutes within the cryogel matrix [22]. Overcoming these diffusion restrictions is crucial for effective operation in real-world applications.

Furthermore, ensuring cryogels’ biocompatibility, minimizing potential toxicity or immunogenicity issues, and conducting extensive biocompatibility testing are essential before utilizing cryogels in practical biomedical applications [23].

Seamless integration of cryogels into existing systems or devices, considering compatibility, proper sealing, connectivity, and overall system performance, is necessary for their practical implementation [25]. Developing cryogels with specific interfaces or ensuring their compatibility with current technologies is important.

Additionally, cost-effectiveness is a significant factor for the widespread use of cryogels in practical applications. Developing efficient manufacturing processes, optimizing the utilization of raw materials, and reducing production costs are essential to make cryogels economically viable.

## 5. Conclusions

Further research is needed to optimize cryogel properties, improve release profiles, and achieve long-term stability. Exploring advanced manufacturing techniques, biomimetic design, and addressing long-term stability and biodegradability are important areas of investigation. Overcoming challenges related to scalability, structural integrity, mass transfer constraints, biocompatibility, seamless integration, and cost-effectiveness is crucial for successful implementation. By addressing these challenges, cryogels can drive the development of innovative biomaterials, revolutionizing biomedical applications.

Disclosure: The authors partly used OpenAI’s large-scale language-generation model. The authors reviewed, revised, and edited the document for accuracy and take full responsibility for the content of this publication. The authors used Bing AI image creator to draw the graphical abstract.

## Data Availability

Not applicable.
